# Reconsidering Mineral Formulations in Human Health and Disease: Molecular Mechanisms, Bioavailability and Metabolic Fates

**DOI:** 10.3390/ijms27209242

**Published:** 2026-10-18

**Authors:** Minsook Ye, Sung Ja Rhie, Youngdae Park, Walter Kim, Insop Shim

**Affiliations:** 1 Department of Physiology, College of Medicine, Kyung Hee University, Seoul 02447, Republic of Korea; 2 Department of Beauty Design, Halla University, Wonju 63092, Republic of Korea; 3 Cosan Co., 709-102, Sansuro, Chowol-Eup, Gwangju-si 12730, Republic of Korea

**Keywords:** organic vs. inorganic minerals, bioavailability, trace elements

## Abstract

Minerals are essential nutrients involved in enzymatic catalysis, signal transduction, cellular bioenergetics, and the maintenance of structural integrity. However, much of the existing research in mineral nutrition has evaluated physiological effects primarily at the elemental level, without adequately distinguishing whether a mineral is ingested as part of an organic matrix in natural foods or enters the biological system as an ionized species from inorganic salts or aqueous solutions. This review examines the criteria by which the conceptual definitions of organic and inorganic minerals have been established and explores the historical, analytical, and methodological factors underlying the lack of clear distinctions between these categories. In addition, it analyzes how the chemical form of minerals governs intestinal transport pathways, systemic bioavailability, tissue-specific accumulation, and intracellular metabolic handling. Using vanadium, silicon, zinc, and selenium as representative paradigms, this review contrasts the distinct molecular and toxicological fates of organic complexes versus inorganic salts-highlighting critical pathways such as protein tyrosine phosphatase (PTPase)-mediated insulin signaling, collagen cross-linking, mitochondrial mitochondrial aconitase (m-aconitase) regulation, and selenoprotein-dependent redox homeostasis. Furthermore, this review addresses the pharmacological and toxicological windows associated with high-dose inorganic salt exposure versus sustained food-bound assimilation. The purpose of this review is not to assert the absolute superiority of one mineral form over another, but rather to clarify poorly defined conceptual boundaries, reassess the interpretative scope of existing findings, and emphasize the necessity for future nutritional and biomedical research to adopt precise, form-specific molecular frameworks.

## 1. Introduction

Minerals are classified as essential nutrients alongside proteins, fats, carbohydrates, and vitamins, executing vital catalytic, structural, and regulatory functions across all biological systems [1]. Calcium and phosphorus form the inorganic scaffold of mineralized tissues and act as secondary messengers in cellular signaling; sodium and potassium maintain transmembrane electrochemical gradients essential for resting potential and secondary active solute transport; and transition elements such as zinc and selenium serve as indispensable catalytic cofactors or structural components within metalloenzymes and antioxidant defense networks [2].

The field of mineral nutrition has historically developed around the identification of deficiency diseases and the establishment of dietary intake recommendations [3]. Within this framework, minerals have primarily been defined and quantified at the elemental level, and relatively limited attention has been given to the chemical forms and coordination environments in which the same element enters the body.

In practice, minerals in foods are commonly present in association with proteins, amino acids, organic acids, or polysaccharides, whereas minerals consumed through water or inorganic salts typically dissociate in aqueous solutions and exist in ionic form [4]. Despite the potential for these differences in chemical form to influence gastric dissolution kinetics, access to distinct apical transport systems (such as divalent cation carriers versus peptide permeases), competitive intestinal uptake, downstream cellular trafficking, and routes of excretion, such distinctions have not been consistently addressed in previous research. In cellular biology and molecular pathology, where precise trace element homeostasis is required for maintaining metabolic integrity and preventing macromolecular damage, understanding these form-dependent disparities is essential for interpreting bioenergetic and toxicological outcomes.

Accordingly, the central question of this review is whether the physiological effects reported in existing mineral studies can be attributed to the intrinsic properties of the elemental minerals themselves, or whether they reflect outcomes that are dependent on specific chemical forms employed in experimental contexts. On the basis of this perspective, the present review re-examines the conceptual boundaries between organic and inorganic minerals, discusses their cellular and biochemical implications, and considers their relevance to molecular pharmacology and metabolic homeostasis.

### 1.1. Scope of the Review

This review qualitatively examines existing experimental and observational studies, including both human and animal research, to assess how the chemical forms in which minerals enter the body have influenced interpretations of bioavailability, metabolic handling, and physiological effects. Rather than conducting a systematic literature review or meta-analysis, this article adopts a narrative review approach to analyze the conceptual and methodological limitations arising from the insufficient differentiation of mineral forms in previous studies, with a specific emphasis on membrane transport mechanisms, intracellular organelle handling, and concentration-dependent toxicological thresholds. The aim of this review is not to determine the superiority of organic versus inorganic minerals, but rather to clarify conceptual boundaries that have remained insufficiently defined, to recalibrate the interpretative scope of existing findings, and to emphasize the need for more precise, form-based approaches in future mineral nutrition and biomedical research. The contribution of the present review lies not in reclassifying the effects of individual minerals, but in systematically revealing the limitations of element-centered interpretations that have been implicitly adopted across mineral research, and in proposing a conceptual framework for form-dependent interpretation within molecular physiology and metabolic medicine.

### 1.2. Operational Definition and Chemical Speciation of “Organic” Minerals

In strict chemical nomenclature, the term “organic mineral” is an operational convention: minerals are fundamentally inorganic elements, and “organic” refers strictly to the coordinating ligand, counterion, or covalent matrix. To eliminate ambiguity, we rigorously delineate this term into four distinct chemical classes:

Organic Acid Salts (Ionic Associations): Weak electrostatic associations (e.g., zinc gluconate, calcium lactate, magnesium citrate). Lacking heterocyclic chelate rings, they dissociate rapidly into solvated free cations under gastric acidolysis (log K < 4).

Coordinate Chelates (True Chelation Complexes): Heterocyclic ring structures formed via coordinate-covalent bonds with electron-donating atoms (N, O, S) of polydentate ligands (e.g., zinc bisglycinate, ferrous bisglycinate). They possess high thermodynamic stability (log β_n_ > 7.0) and largely resist gastric dissociation.

Supramolecular Matrices: Macromolecular assemblies wherein minerals are encapsulated within protein cages or peptide clusters (e.g., ferritin, casein phosphopeptides), protecting the metal center via steric shielding.

Covalent Biomimetics (Organometallics): True carbon–heteroatom covalent bonds (e.g., L-selenomethionine) that mimic canonical metabolites and are assimilated via amino acid transport pathways. Throughout this review, the ambiguous umbrella term “organic minerals” is replaced with these specific speciation descriptors.

## 2. Conceptual Definitions of Organic and Inorganic Minerals

### 2.1. Organic Minerals and Food-Bound Forms

Organic nutrients are characterized by carbon-based covalent structures and participate in biological processes while retaining their molecular forms. Proteins, lipids, carbohydrates, and vitamins are representative examples of organic nutrients. In foods, minerals are often present in association with such organic matrices, rather than as isolated inorganic elements, and may therefore function within a distinct biological context [5].

Conceptually, organic minerals may be classified into two broad categories. The first includes minerals that naturally occur in foods bound to proteins, amino acids, organic acids, or polysaccharides, where they are integrated as part of the structural or functional components of the food matrix. The second category comprises synthetic organic mineral forms used in supplements, such as amino acid or organic acid chelates. Mechanistically, the mucosal uptake of organic mineral forms is governed by their thermodynamic stability constants (log K) and gastric survival kinetics. Experimentally verified bidentate chelates with high stability constants (e.g., zinc bisglycinate [logβ2 ≈ 8.2] and ferrous bisglycinate [logβ2 ≈ 7.5]) can largely resist gastric acidolysis, remaining partially intact to utilize specialized apical peptide transport pathways (such as PepT1/SLC15A1) or fluid-phase endocytosis, as demonstrated in competitive inhibition assays with glycyl-sarcosine (Gly-Sar) in human Caco-2 monolayers [6,7,8]. This neutral coordinate shielding reduces premature precipitation with dietary antagonists (e.g., phytates) and moderates non-specific membrane interactions. In contrast, moderately stable organic complexes or organic acid salts (e.g., citrates, gluconates, and aspartates; log K < 4.0) undergo partial or complete ligand dissociation across the gastrointestinal pH gradient, releasing solvated divalent cations that are subsequently absorbed via canonical divalent metal permeases (such as DMT1/SLC11A2 and ZIP4/SLC39A4) [9,10,11,12].

### 2.2. Dynamic Speciation Across Gastrointestinal pH Gradients: Thermodynamic Stability

Constants and Ligand Exchange to avoid an idealized model of intact organic mineral permeation, mucosal handling must be evaluated against the dynamic physicochemical transitions of the human digestive tract. Following oral ingestion, mineral complexes traverse a steep luminal pH gradient, shifting from the strongly acidic gastric environment (pH 1.5–2.0) to the neutralized proximal duodenum (pH 5.5–6.5) and slightly alkaline jejunum/ileum (pH 6.8–7.4). Under gastric acidolysis, high hydronium ion concentrations (H^+^) challenge coordinate metal-ligand bonds. The extent of gastric dissociation is fundamentally governed by the conditional stability constant (logK) and the protonation constant (pK_a_) of the coordinating ligand:ML_2_ + 2H^+^ →M^2+^ + 2HL Organic acid salts and weakly coordinated complexes with low thermodynamic stability constants (log K < 4, e.g., zinc gluconate, magnesium citrate, and calcium lactate) undergo near-total ligand dissociation in the gastric lumen, releasing solvated divalent cations. Conversely, bidentate amino acid chelates possessing high cumulative stability constants (logß_2_ ≈ 7.5–8.5, e.g., ferrous bisglycinate and zinc bisglycinate) exhibit thermodynamic resistance to acid-driven dissociation, retaining significant coordinate integrity through gastric transit. As chime empties into the duodenum, neutralization by pancreatic bicarbonate induces a dynamic phase of competitive ligand exchange. Free or partially dissociated cations are exposed to a complex milieu of endogenous ligands (e.g., mucins, bile acids, free digestive enzymes) and dietary antagonists (e.g., phytates, polyphenols, inorganic orthophosphates). At this juncture, unshielded inorganic ions readily form insoluble, high-lattice-energy precipitates (such as insoluble zinc or ferric phytate complexes) that are eliminated unabsorbed in feces. In contrast, minerals embedded in natural supramolecular food matrices (e.g., ferritin nanocages, selenomethionine in native proteins, or milk phosphopeptides) or kinetically robust synthetic chelates are protected from rapid precipitation, undergoing gradual, controlled dissociation or intact endocytic/transporter-mediated mucosal entry. Thus, intestinal bioavailability is not a binary consequence of “organic” versus “inorganic” labeling, but a kinetic manifestation of coordination thermodynamic equilibria, ligand exchange competition, and luminal pH dynamics (Table 1).

### 2.3. Inorganic Minerals and Ionic Forms

**Figure 1:**
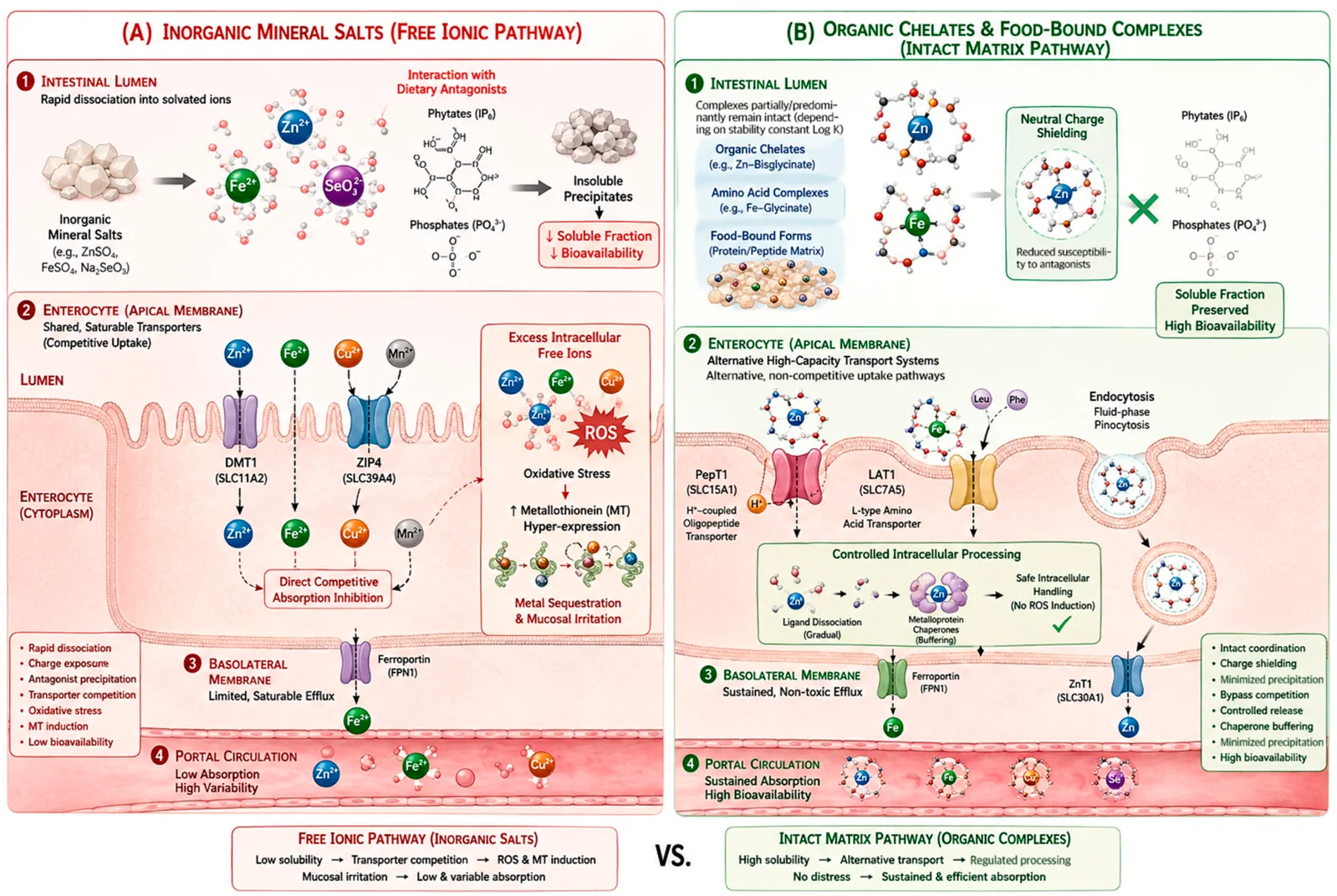
Relative Tendencies in Intestinal Processing and Enterocyte Handling: Inorganic Mineral Salts versus Organic Coordination Complexes. (**A**) Inorganic Mineral Salts (Free Ionic Pathway Tendencies): In the intestinal lumen, inorganic salts rapidly dissociate into solvated, charged cations and anions. These unshielded ions exhibit a higher propensity to precipitate upon interacting with dietary antagonists such as phytates and phosphates, often reducing the bioavailable soluble fraction. Solubilized free cations enter enterocytes primarily via shared, saturable apical transport systems including Divalent Metal Transporter 1 (DMT1/SLC11A2) and Zrt-/Irt-like protein 4 (ZIP4/SLC39A4), provoking direct competitive absorption inhibition against other essential trace elements (e.g., copper and iron). Excessive intracellular surges of free ionic species can elevate localized mucosal oxidative stress and induce metallothionein hyperexpression, which sequesters entering metals and may provoke mucosal irritation. (**B**) Organic Chelates & Food-Bound Complexes (Coordinate Pathway Tendencies): Coordinate complexes and food matrix-integrated forms exhibit varying degrees of thermodynamic stability (log K). Highly stable bidentate chelates largely retain their coordination integrity across physiological pH gradients, whereas weaker complexes undergo partial luminal dissociation (M-L_2−_ ≡ M^2^ + 2L^−^). Neutral charge shielding generally reduces insoluble complexation with luminal antagonists. Stable coordination complexes mitigate competitive divalent cation transporter saturation by utilizing alternative or parallel apical transport pathways, including proton-coupled oligopeptide transporters (PepT1/SLC15A1) or amino acid transport systems (LAT1/SLC7A5). Following gradual intracellular ligand dissociation and metalloprotein chaperone buffering, the micronutrients undergo sustained basolateral transfer into the portal circulation with moderated mucosal oxidative distress. (Note: The illustrated pathways represent conceptual physicochemical models and relative tendencies under physiological conditions, rather than immutable, uniform biological absolutes across all organic mineral formulations).

In contrast, minerals consumed through water or inorganic salts dissociate in aqueous solutions and exist predominantly in ionic form, exerting their biological effects through electrochemical properties that are essential for nerve transmission, muscle contraction, osmotic regulation, and the modulation of enzyme activity [13]. Although these inorganic ions are indispensable for physiological function, they lack defined molecular structures or storage forms, and may therefore exhibit biological behaviors distinct from minerals bound within organic matrices. Unbound mineral cations readily interact with shared divalent metal transporters, rendering them susceptible to competitive transport inhibition and increasing the propensity for localized reactive oxygen species generation when present in excess. Much of the existing mineral research has interpreted experimental findings without clearly distinguishing these differences in chemical form, a practice that has contributed to the simplification of mineral function at the elemental level (Figure 1).

### 2.4. Intracellular Ligand Cleavage and the Functional Rationale for Organic Coordination

A critical question in mucosal mineral handling concerns the ultimate cellular fate of intact coordination complexes: rather than persisting as inert, permanently encapsulated entities within systemic circulation, internalized chelates undergo rapid and complete intracellular dissociation to enter the functional metabolic pool. Following apical mucosal uptake via proton-coupled oligopeptide permeases such as PepT1 (SLC15A1) or via clathrin-dependent macropinocytosis, the coordinate bonds are dismantled through coordinated cytosolic and vesicular pathways. Enterocytic cytosolic aminopeptidases, dipeptidases, and intracellular esterases rapidly hydrolyze the coordinating amino acid or peptide backbones, destabilizing the heterocyclic ring structure. Concurrently, complexes internalizing through endosomal compartments encounter progressive vesicular acidification (pH 4.5–5.5), which promotes hydronium-driven protonation of coordinating heteroatoms and induces metal cation dissociation. Liberated cations are subsequently intercepted by high-affinity intracellular metallochaperones, including metallothionein for zinc and copper, ATOX1, and poly-rC-binding proteins for iron, which direct the essential trace elements toward cellular apo-enzymes, storage proteins, or basolateral exporters such as ferroportin 1 and ZnT1. Consequently, the definitive biological advantage of organically bound minerals does not reside in permanent systemic chelation, but rather in their function as transient, protective molecular shuttles across the hostile luminal milieu of the upper gastrointestinal tract. By coordinating the central metal atom within an internal ring architecture, robust chelates provide charge neutralization and steric shielding that effectively prevent premature lattice precipitation with dietary antagonists such as phytates, polyphenols, and orthophosphates, which otherwise eliminate unshielded inorganic salts as unabsorbable fecal complexes. Moreover, by accessing parallel, high-capacity peptide transport systems and fluid-phase endocytosis, organic complexes circumvent the saturable, competitively inhibited apical metal permeases—specifically DMT1 (SLC11A2) and ZIP4 (SLC39A4)-thereby preventing mutual trace-element antagonism and maintaining high uptake rates even under mineral-replete or antagonist-rich conditions. Finally, the sequestration of free cationic charges during transit minimizes direct catalytic initiation of mucosal Fenton chemistry and suppresses hyperosmotic epithelial stress, drastically diminishing the acute mucosal irritation, nausea, and cramping that frequently compromise patient adherence to inorganic mineral salts.

### 2.5. Coexistence of Definition Criteria

In the existing literature, organic and inorganic minerals have been defined according to different criteria, including bioavailability, physiological function, and chemical composition [4]. Some studies have attempted to distinguish these categories on the basis of relative absorption efficiency; however, substantial variability in bioavailability has been repeatedly reported even among mineral forms classified within the same organic category [4]. Other approaches have relied on criteria such as tissue storage, metabolic fate, or functional roles, yet these do not provide explicit definitions grounded in chemical form.

The most traditional distinction is based on chemical composition, differentiating minerals bound to organic compounds from those supplied as inorganic salts [14]. Nevertheless, even within the same chemical category, minerals may display divergent biological behaviors, limiting the extent to which physiological effects can be generalized.

Taken together, the coexistence of these definition criteria suggests that the concepts of organic and inorganic minerals should not be understood along a single axis, but rather within a multidimensional framework encompassing chemical structure, thermodynamic stability, apical transport kinetics, and functional context.

### 2.6. Limitations of the Organic–Inorganic Dichotomy in Existing Literature

Numerous review articles have noted that the binary distinction between organic and inorganic minerals is insufficient to consistently explain differences in mineral bioavailability or physiological effects. Comprehensive evaluations comparing the relative bioavailability of trace minerals have reported substantial variability among mineral forms classified as organic, indicating that categorical classification alone cannot adequately account for observed outcomes. These studies have also highlighted inconsistencies in how organic and inorganic minerals are defined across different investigations, suggesting that chemical bonding characteristics and functional properties may be more informative for interpretation than dichotomous classification.

Other reviews have raised concerns regarding the lack of standardized definitions in studies comparing organic and inorganic mineral sources. The term “organic mineral” has been used to collectively describe chemically diverse forms, including amino acid chelates, proteinates, and organic acid complexes, while “inorganic mineral” encompasses a wide range of salts such as sulfates, oxides, and chlorides. This diversity in terminology complicates direct comparisons among studies and limits the derivation of generalized conclusions based solely on organic versus inorganic classification.

Findings from systematic reviews and meta-analyses have reached similar conclusions, indicating that categorizing mineral sources as organic or inorganic alone does not reliably predict changes in physiological responses or metabolic indicators. Rather, mineral effects appear to be influenced by interactions among chemical form, dosage, physiological status, and experimental context.

Collectively, these observations suggest that while the organic–inorganic distinction may serve as a useful starting point for study design, it has inherent structural limitations in explaining mineral bioavailability and physiological function. Future research in mineral nutrition will therefore require approaches that move beyond categorical classification to explicitly differentiate mineral forms according to their chemical, functional, and mechanistic characteristics.

## 3. Case Studies Illustrating Form-Dependent Interpretation

To illustrate how mineral form has influenced the interpretation of physiological effects in existing research, this section reviews representative case studies while explicitly considering the chemical forms of minerals employed in experimental and human studies. By examining the correspondence between observed effects and the mineral forms used, this section highlights how definitions of organic and inorganic minerals have been implicitly applied in the interpretation of previous findings, and addresses their specific impact on cellular bioenergetics, enzymatic kinetics, and organ-specific toxicological pathways.

### 3.1. Vanadium

Vanadium has not been formally classified as an essential mineral; however, it has attracted research interest due to reported physiological effects related to the regulation of glucose metabolism [15]. Studies using streptozotocin-induced diabetic rodent models have shown that administration of vanadyl sulfate or other inorganic vanadium salts results in reduced blood glucose levels, improved insulin sensitivity, and enhanced glucose tolerance [16]. These studies reported significant improvements across multiple metabolic indices, including fasting blood glucose, postprandial glucose responses, and peripheral glucose utilization [16]. In some cases, such effects were observed in the absence of exogenous insulin administration, leading to the interpretation that vanadium compounds may directly or indirectly mimic insulin signaling [17].

Several studies further reported that supplementation with inorganic vanadium salts elicited pronounced hypoglycemic responses in diabetic models. Mechanistic investigations have attributed these effects to the inhibition of protein tyrosine phosphatases and the activation of downstream insulin receptor signaling pathways. Specifically, vanadium compounds have been shown to stimulate the PI3K-Akt signaling pathway, promote translocation of glucose transporter 4 (GLUT4) to the cell membrane, suppress hepatic gluconeogenesis, and enhance glucose oxidation in skeletal muscle [18]. These molecular cascades reflect direct phosphatase inhibition by high intracellular concentrations of orthovanadate or vanadyl ions rather than physiological cofactor modulation.

**Figure 2:**
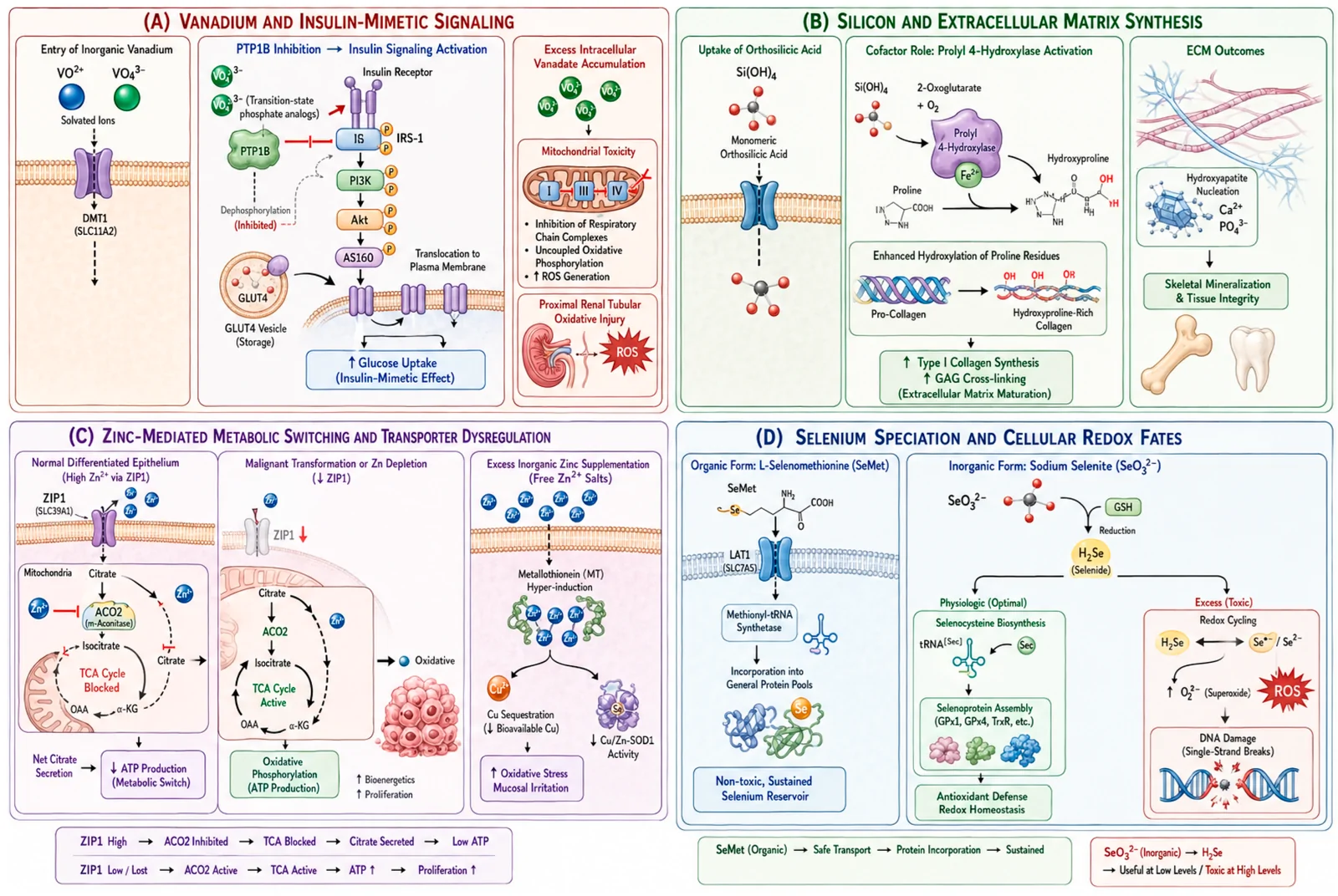
Form-Dependent Intracellular Signaling Pathways, Metabolic Regulation, and Organelle-Specific Fates. (**A**) Vanadium and Insulin-Mimetic Signaling: Solvated inorganic vanadyl and orthovanadate ions act as transition-state phosphate analogs, directly inhibiting protein tyrosine phosphatase 1B. This sustains phosphorylation of insulin receptor substrate 1 and downstream cascades, promoting the translocation of glucose transporter 4 storage vesicles to the plasma membrane independently of exogenous insulin. In contrast, excess intracellular accumulation of free vanadate uncouples mitochondrial oxidative phosphorylation, provoking respiratory chain complex inhibition and proximal renal tubular oxidative injury. (**B**) Silicon and Extracellular Matrix Synthesis: Monomeric orthosilicic acid is passively assimilated into osteoblasts and fibroblasts, where it does not serve as a classical catalytic cofactor-given that prolyl 4-hydroxylase canonically requires Fe(II), 2-oxoglutarate, and ascorbate-but instead functions as a metabolic and transcriptional modulator that stimulates prolyl 4-hydroxylase expression and activity. This enzymatic stimulation enhances the hydroxylation of proline residues, stimulating pro-collagen maturation, type I collagen synthesis, and glycosaminoglycan cross-linking within the extracellular matrix, which facilitates targeted hydroxyapatite nucleation and skeletal mineralization. (**C**) Zinc-Mediated Metabolic Switching and Transporter Dysregulation: In well-differentiated epithelial tissues (e.g., normal prostate epithelium), high intracellular zinc accumulation driven by the apical influx transporter ZIP1 (SLC39A1) potently inhibits mitochondrial m-aconitase. This blocks the conversion of citrate to isocitrate, truncating the tricarboxylic acid cycle and shifting cellular bioenergetics toward net citrate secretion rather than adenosine triphosphate (ATP) synthesis. Malignant cellular transformation or severe cellular zinc depletion down-regulates ZIP1, relieving m-aconitase inhibition and reactivating oxidative phosphorylation to fuel rapid tumor proliferation. Exogenous over-supplementation with free inorganic zinc salts provokes mucosal metallothionein hyper-induction in enterocytes, which avidity-sequesters dietary copper, arresting its basolateral export and provoking secondary systemic copper deficiency. (**D**) Selenium Speciation and Cellular Redox Fates: Organic L-selenomethionine utilizes neutral amino acid transporters and is non-specifically incorporated into general somatic protein pools via methionyl-tRNA synthetase, serving as an amino acid reservoir that supports selenoprotein synthesis under physiological demand, but carries liabilities of unregulated proteomic accumulation and metabolic perturbation during chronic supra-nutritional intake. In contrast, inorganic sodium selenite undergoes rapid, glutathione-dependent reduction to free hydrogen selenide. While physiologic hydrogen selenide serves as the obligate substrate for selenocysteine biosynthesis and antioxidant selenoprotein assembly, excessive free selenide enters rapid redox cycling, generating superoxide anions (O_2_^•−^) and precipitating genomic single-strand breaks.

However, these physiological effects have been derived predominantly from experimental conditions involving inorganic vanadium salts. Crucially, a clear distinction must be established between basal nutritional exposure and supra-physiological pharmacological interventions. Under normal physiological conditions, vanadium functions as an ultra-trace element; human daily dietary intake typically ranges between 10 and 30 μg/day (largely derived from whole grains, mushrooms, and shellfish), maintaining baseline circulating concentrations in the sub-nanomolar to low nanomolar range (<0.5–2nM). In stark contrast, documented antidiabetic and insulin-mimetic responses in rodent models have relied on pharmacological administration of inorganic salts (such as vanadyl sulfate or sodium orthovanadate) at 0.1 to 1.0 mmol/kg/day (approximately 15–100 mg elemental V/kg/day), which elevates intracellular tissue concentrations into the micromolar range (5–50 μg M). Similarly, human diabetic clinical trials have employed vanadyl sulfate doses of 50 to 150 mg/day (roughly 15–45 mg elemental V/day), exceeding baseline dietary intake by more than 1000-fold. Existing literature has noted the near absence of comparative studies that reflect the chemical forms of vanadium as consumed in natural foods. In addition, several studies have reported adverse effects associated with high-dose inorganic vanadium supplementation, including mitochondrial respiratory chain impairment, proximal renal tubular cytotoxicity, and gastrointestinal irritation. Because the micromolar concentrations required for PTPase inhibition directly overlap with these cytotoxic thresholds, inorganic vanadium compounds exhibit an exceptionally narrow therapeutic window. Consequently, some authors have argued that the metabolic effects observed for vanadium are more appropriately interpreted as pharmacological actions arising from the chemical properties and high biological reactivity of specific inorganic vanadium compounds at supra-physiological levels, rather than as physiological functions of an essential dietary mineral. In this regard, organic oxovanadium coordination complexes (such as bis(maltolato)oxovanadium(IV), BMOV) have been shown to enhance membrane permeability and lower effective elemental dosing requirements, thereby mitigating renal and gastrointestinal cytotoxicity compared to free inorganic salts. Therefore, evaluating vanadium strictly at the elemental level without accounting for dosing thresholds, speciation, and the narrow therapeutic index of free inorganic vanadium species remains a significant limitation in metabolic research (Figure 2A).

### 3.2. Silicon

Silicon is a trace element for which potential roles in connective tissue and bone formation have been proposed. Carlisle reported that silicon deficiency induced abnormalities in skeletal development in chick models [19]. Subsequent studies using rodent models observed that supplementation with silicon in the form of silicic acid improved indices related to bone mineral density and collagen synthesis [20]. Mechanistically, dietary silicon is absorbed across the intestinal brush border predominantly as neutral monomeric orthosilicic acid (Si(OH)_4_) via aquaporin-like permeases and solute carriers functionally homologous to nodulin-26-like intrinsic channels (including AQP9 and SLC4 exchangers) before capillary diffusion [21]. Within osteoblasts and fibroblasts, orthosilicic acid functions as a metabolic co-activator that upregulates prolyl-4-hydroxylase expression, promoting proline hydroxylation to stabilize type I collagen triple helices and extracellular matrix cross-linking [22]. However, silicon used in most experimental studies has been supplied predominantly in inorganic forms, specifically monomeric orthosilicic acid or synthetic silica nanoparticles. Several authors have noted the limited availability of direct comparative data between inorganic silicon and phytosilicate forms naturally present in foods [23]. Human studies have likewise focused primarily on observational associations between total silicon intake and bone mineral density, limiting the ability to establish causal relationships regarding differences in bioavailability or metabolic pathways according to chemical form [24].

Consequently, while silicon is frequently implicated in osteoblast proliferation and extracellular matrix stability, current clinical interpretations rely on total intake data rather than form-dependent efficacy, leaving the cellular uptake kinetics and biological fates of food-derived phytosilicates versus inorganic silicates largely unverified (Figure 2B).

### 3.3. Zinc

Zinc represents a mineral for which differences in bioavailability according to chemical form have been relatively well documented. Human studies have reported that organic chelated forms of zinc, in which zinc is bound to amino acids or organic acids, exhibit higher absorption efficiency and lower gastrointestinal irritation compared with inorganic zinc salts such as zinc sulfate [25]. From a molecular transport perspective, organically bound zinc exhibits dual absorption kinetics: while partially dissociated ionic fractions utilize canonical ZIP4 (SLC39A4) and DMT1 permeases, persistent intact chelates engage parallel, non-competitive uptake pathways via peptide transporters (such as PepT1) or fluid-phase endocytosis, thereby mitigating apical saturation bottlenecks and competitive inhibition.

At the intestinal apical membrane, bidentate zinc bisglycinate possesses high thermodynamic stability (logβ_2_ ≈ 8.2), remaining largely intact to utilize the proton-coupled oligopeptide transporter PepT1 (SLC15A1) in parallel to canonical ZIP4 permeases, as confirmed by competitive inhibition assays with glycyl-sarcosine (Gly-Sar) in Caco-2 monolayers [7,8]. Furthermore, macromolecular food-bound zinc complexes can access fluid-phase endocytosis, avoiding apical transporter competition under high dietary antagonist loads [26]. These transport kinetics reinforce that oral mineral bioavailability is governed by coordination chemistry and formulation properties rather than total elemental mass alone [27]. Following basolateral transfer into systemic circulation, zinc exhibits distinct, tissue-specific metabolic handling across specialized cell types [28]. In normal prostate epithelial cells, zinc ions are actively accumulated at high intracellular concentrations to potently inhibit the mitochondrial m-aconitase enzyme. Because m-aconitase catalyzes the stereospecific isomerization of citrate to isocitrate within the tricarboxylic acid (TCA) cycle, blocking this specific enzymatic step causes cells to accumulate citrate for secretion rather than oxidizing it through oxidative phosphorylation for adenosine triphosphate (ATP) production [29]. This distinct metabolic architecture represents an evolutionary optimization that supports tissue-specific secretory functions, despite a resulting decrease in local bioenergetic efficiency.

Conversely, a hallmark of malignant transformation in these epithelial cells is the systematic down-regulation of primary zinc transporters, such as ZIP1 (SLC39A1), which leads to a severe depletion of intracellular zinc levels, the reactivation of m-aconitase, and an accelerated metabolic shift toward coupled TCA cycle flux and aerobic glycolysis to sustain cellular proliferation [30]. Although tissue-specific zinc deficiency serves as a prominent metabolic marker for cellular dysfunction, clinical and epidemiological efforts to alter or normalize these internal mineral profiles through exogenous high-dose inorganic supplementation have frequently resulted in systemic adverse effects rather than targeted therapeutic outcomes.

Epidemiological data, most notably from the Health Professionals Follow-up Study (HPFS) which longitudinally tracked 47,240 men over a 28.3-year period, demonstrate that long-term, high-dose zinc supplementation exceeding 75 mg/day significantly increases the hazard ratio (HR) for aggressive, fatal prostate complications (HR: 1.76; 95% CI: 1.16–2.66), with a similar escalation observed in cohorts maintaining supplementation for over 15 years (HR: 1.91; 95% CI: 1.28–2.85). This outcome is largely driven by disrupted systemic trace metal homeostasis: uncalibrated intake of free inorganic zinc salts induces mucosal metallothionein overexpression in enterocytes, which irreversibly sequesters dietary copper, provoking systemic secondary copper deficiency, impairment of Cu/Zn-superoxide dismutase (SOD1) activity, and subsequent baseline oxidative DNA damage. In addition, excessive circulating zinc can alter steroid hormone receptor dynamics and peripheral endocrine signaling.

These findings highlight that attempting to correct localized cellular zinc deficits using high boluses of inorganic salts triggers counter-regulatory and toxicological responses. Inorganic zinc salts dissociate rapidly in the gastrointestinal lumen, saturating carrier-mediated transport and precipitating localized mucosal irritation. Conversely, robust coordinate zinc chelates mitigate acute mucosal distress and bypass carrier saturation under meal-associated antagonist challenges; however, at prolonged supra-nutritional dosages, both organic and inorganic forms release systemic elemental zinc that drives intestinal metallothionein overexpression and secondary copper depletion.

Despite these clear biological distinctions, a substantial portion of existing nutritional research remains limited by a narrow focus on short-term absorption kinetics and transient fluctuations in circulating serum levels. Definitive empirical evidence regarding long-term variations in tissue distribution, intracellular storage patterns, and targeted physiological function according to specific zinc coordination complexes remains sparse. Consequently, current findings indicate that while the core physiological roles of zinc are not fundamentally altered at the elemental level, the metabolic efficiency, systemic safety, and tissue-specific handling of the mineral are entirely modulated by the chemical form in which it is supplied. Within molecular pharmacology and cellular nutrition, differences among zinc compounds must be interpreted as structural determinants of membrane transport kinetics, ligand exchange dynamics, and systemic safety margins, rather than as binary determinants of the presence or absence of elemental function (Figure 2C).

### 3.4. Selenium

Selenium is a mineral for which form-dependent differences in biological handling have been reported with relative consistency. Organic selenium forms, such as selenomethionine and selenium-enriched yeast, are incorporated into proteins and function as storage forms that provide stable precursors for selenoprotein synthesis. In contrast, inorganic selenium forms, such as sodium selenite, are more rapidly metabolized and have been reported to enter toxic pathways at higher doses [31]. At the molecular level, selenomethionine is recognized by methionyl-tRNA synthetase and non-specifically incorporated into the general somatic protein pool via neutral amino acid transport systems (e.g., LAT1), creating a reversible intracellular reservoir. Conversely, inorganic selenite (SeO_3_^2−^) is absorbed via non-specific anion mechanisms and undergoes immediate reduction by intracellular glutathione (GSH) and thioredoxin systems, generating hydrogen selenide (H_2_Se). While hydrogen selenide is the requisite substrate for selenophosphate synthetase 2 in de novo selenocysteine biosynthesis, excess accumulation of free selenide rapidly undergoes redox cycling, generating superoxide anions (O_2_) and inducing DNA single-strand breaks.

**Figure 3:**
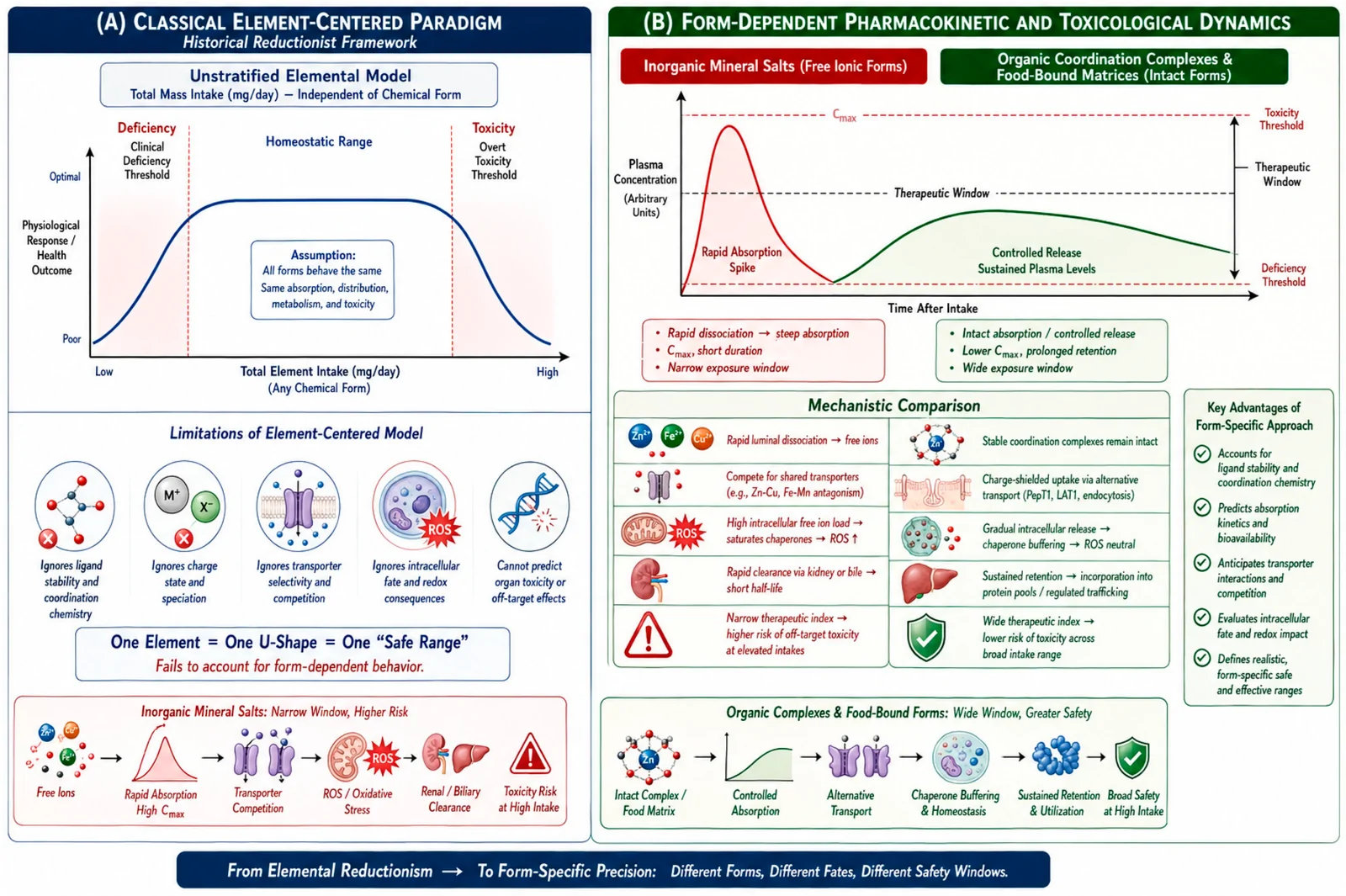
Conceptual Transition from Elemental Reductionism to Form-Specific Pharmacokinetic and Toxicological Windows. (**A**) Classical Element-Centered Paradigm: Historical dietary models operate on an unstratified, elemental basis (total mass intake; mg/day), assuming a uniform U-shaped homeostatic curve bounded simply by clinical deficiency and overt toxicity thresholds. This reductionist framework fails to account for ligand stability, charge states, or coordination chemistry. (**B**) Form-Dependent Pharmacokinetic and Toxicological Dynamics: Inorganic Mineral Salts (Red Curve): Characterized by rapid luminal dissociation and steep systemic absorption spikes (C max). Free ionic surges saturate physiological chaperone pools, trigger competitive transporter cross-talk (e.g., Zn-Cu antagonism), and elicit acute intracellular ROS generation. Because unbound ions are rapidly cleared via renal filtration or biliary excretion, inorganic forms exhibit a narrow therapeutic index and an elevated risk of off-target tissue toxicity at high intakes. Organic Coordination Complexes & Food-Bound Matrices (Green Curve): Characterized by controlled release kinetics, charge-shielded intestinal absorption, and sustained plasma retention. Intact coordination complexes integrate into endogenous protein reservoirs or regulated intracellular.

Through the activity of selenoproteins, including glutathione peroxidase (GPx) and thioredoxin reductases (TrxR), selenium contributes to the mitigation of oxidative stress and suppression of genomic instability. Several epidemiological studies have reported associations between selenium status and the risk or progression of prostate-related disorders and broader degenerative pathologies. Accordingly, selenium has been relatively consistently recognized in the literature as a mineral for which chemical form influences the durability of physiological effects, patterns of tissue storage, and safety profiles [32]. However, the systemic safety and efficacy of selenium are heavily governed by its baseline status and chemical speciation, as demonstrated by large-scale clinical investigations. The Selenium and Vitamin E Cancer Prevention Trial (SELECT), which evaluated a cohort of over 35,000 participants, illustrated that untargeted selenium supplementation (200 μg/day as L-selenomethionine) failed to provide systemic protective benefits and, in individuals with high baseline selenium levels, significantly increased the risk of high-grade disease. This underscores a U-shaped dose–response curve where excessive administration in a state of physiological repletion induces paradoxical cellular alterations, including dysregulation of cellular protein folding machinery and aberrant redox signaling. Inorganic selenite presents a substantially narrower therapeutic index compared to organic forms due to its direct catalytic generation of reactive intermediates prior to elimination. Crucially, the avoidance of acute mucosal irritation and redox cycling by organic forms must not be conflated with absolute toxicological safety. While organic L-selenomethionine bypasses apical mucosal irritation, chronic supra-nutritional exposure saturates the somatic protein pool, leading to unregulated tissue retention. Prolonged turnover of this non-specific reservoir liberates sustained loads of selenide that can trigger endoplasmic reticulum stress, aberrant protein folding, impaired insulin signaling, and elevated risks of type 2 diabetes mellitus alongside classic chronic selenosis. Therefore, while organic complexes mitigate acute gastrointestinal distress, they inherently carry risks of insidious, cumulative tissue burden and chronic metabolic perturbation upon persistent over-supplementation (Figure 3). Consequently, selenium represents a clear illustration of how chemical form determines the safety profile and physiological durability of mineral interventions (Figure 2D).

## 4. A Biophysical Framework and Classification Matrix for Mineral Speciation

Historically, mineral nutrition research has operated largely under elemental reductionism, evaluating total elemental mass while overlooking chemical speciation and binding geometry [4]. This aggregate approach has generated conflicting findings regarding bioavailability, mucosal tolerance, and long-term metabolic safety across both clinical and toxicological settings. To transcend these descriptive limitations, this section establishes a unified biophysical framework: first, generalizing the speciation principles demonstrated by our focal archetypes to major dietary elements (iron, calcium, and magnesium); second, articulating a four-class predictive matrix based on thermodynamic stability and transport kinetics; and third, delineating the fundamental kinetic and regulatory divergence between intact food matrices and isolated synthetic chelates.

### 4.1. Generalization to Major Macro- and Microminerals: Iron, Calcium, and Magnesium

The physicochemical principles established through our four focal archetypes apply broadly across human mineral nutrition, as illustrated by the distinct speciation behaviors of iron, calcium, and magnesium. For iron (Fe), unshielded inorganic ferrous sulfate (FeSO_4_) readily dissociates in the intestinal lumen, where free Fe^2+^ provokes localized mucosal oxidative distress via Fenton-mediated reactive oxygen species generation and saturates apical DMT1 permeases, competitively impairing the co-absorption of zinc and copper [33,34]. In contrast, bidentate ferrous bisglycinate possesses high thermodynamic stability (logß_2_ ≈ 7.5), mitigating luminal precipitation with dietary polyphenols and phytates while utilizing alternative apical uptake pathways (including PepT1 and fluid-phase endocytosis) to deliver elemental iron with attenuated gastrointestinal irritation [9,35].Similarly, for macrominerals such as calcium (Ca) and magnesium (Mg), simple inorganic salts (e.g., calcium carbonate, magnesium oxide) depend heavily on gastric acid solubilization (CaCO_3_ + 2HCl → CaCl_2_ + H_2_O + CO_2_), leaving unabsorbed ionic fractions that precipitate with phosphates or exert luminal hyperosmotic loads that provoke loose stools and abdominal cramping. Conversely, organic coordination forms such as calcium citrate malate and magnesium bisglycinate exhibit superior aqueous solubility across neutral-to-alkaline luminal pH gradients, facilitating sustained passive paracellular diffusion across the claudin-rich tight junctions of the jejunum and ileum. These parallel behaviors confirm that thermodynamic coordination stability, charge neutralization, and transporter diversification govern mineral bioavailability and mucosal safety across the entire nutritional periodic table.

### 4.2. A Predictive Classification Matrix and Decision Framework for Mineral Speciation

To guide researchers and formulators beyond descriptive organic–inorganic nomenclature, we propose a standardized decision matrix categorizing supplemental and dietary minerals across three biophysical axes: coordination stability (logK), susceptibility to gastrointestinal pH transitions, and operative enterocyte uptake pathways.

Class I: Readily Dissociative Salts (Ionic Lattice/Labile Complexes): Characterized by complete ionic lattice dissolution or weak association constants. These forms yield free solvated cations in gastric juice, rely strictly on canonical saturable permeases (DMT1/SLC11A2, ZIP4/SLC39A4, TRPV6), and exhibit high vulnerability to antagonist precipitation (phytates, polyphenols).

Class II: Robust Coordinate Chelates (High Thermodynamic Stability): Characterized by multi-dentate chelation, neutral charge shielding, and high cumulative stability constants. These complexes largely resist gastric acidolysis, avoid antagonist precipitation, and utilize parallel peptide transporters (PepT1/SLC15A1) or fluid-phase endocytosis.

Class III: Macromolecular/Supramolecular Matrices: Comprising core–shell biomineral architectures (e.g., ferritin nanocages, casein phosphopeptide complexes). Cellular assimilation proceeds via clathrin-dependent macropinocytosis or slow luminal matrix deconstruction, shielding the mineral core against premature lattice formation. Class IV: Covalent Biomimetic Organometallics: Defined by direct carbon-heteroatom covalent bonds (e.g., L-selenomethionine). Absorbed efficiently via system L neutral amino acid transporters (LAT1/LAT2) and non-specifically pooled into the somatic proteome, requiring strict monitoring of cumulative chronic intakes to avoid metabolic disruption.

### 4.3. Natural Food-Bound Matrices Versus Isolated Synthetic Chelates: Mechanistic Kinetics and Regulatory Divergence

A rigorous translational understanding of mineral speciation requires untangling the profound divide between native food-bound minerals and isolated synthetic coordination chelates. In whole foods, minerals are intrinsically embedded within intricate, macromolecular food matrices—exemplified by iron within multimeric ferritin nanocages, zinc complexed within dietary phytate-protein networks, or calcium associated with colloidal casein micelles. The physiological assimilation of these food-bound minerals is fundamentally rate-limited by upstream gastrointestinal transit, gastric mastication, and luminal enzymatic proteolysis, creating a continuous, low-concentration release profile that protects mucosal surfaces and integrates with homeostatic feedback systems. Conversely, orally administered synthetic coordination chelates (e.g., purified zinc or ferrous bisglycinate) deliver concentrated, immediate stoichiometric boluses directly to enterocytes. Because these synthetic small-molecule complexes rely on parallel peptide permeases (PepT1) or rapid fluid-phase pinocytosis, they bypass both gastric enzymatic degradation and apical divalent metal saturation gates.

This kinetic divergence mandates fundamentally distinct regulatory frameworks and toxicological surveillance. Food-bound minerals are ingested as integral dietary components, where the sheer physical bulk and caloric density of the food matrix provide intrinsic satiety-driven safeguards against acute mineral overload. Consequently, their intake is managed via population-level Recommended Dietary Allowances (RDA) without necessitating pre-market toxicological clearance. In sharp contrast, isolated synthetic chelates possess high elemental density, enhanced membrane permeability, and widespread availability in concentrated capsule or tablet formulations. Regulators (such as the European Food Safety Authority and the US Food and Drug Administration) appropriately subject synthetic chelates to novel dietary ingredient or chemical additive safety evaluations, demanding rigorous evidence that the coordinating ligand (e.g., amino acid derivatives, synthetic chelators) does not alter xenobiotic clearance or drive cumulative tissue accumulation. Recognizing that synthetic chelates behave kinetically more akin to pharmacological prodrugs than to native dietary food matrices is essential for reconciling clinical bioavailability efficacy with long-term toxicological safety.

## 5. Historical Evolution and Modern Analytical Platforms in Mineral Speciation

The insufficient development of clear conceptual distinctions regarding mineral forms reflects not merely a theoretical gap, but is closely linked to the historical trajectory of mineral nutrition research and to methodological constraints in experimental practice.

### 5.1. Element-Centered Origins of Mineral Nutrition

During the late nineteenth and early twentieth centuries, nutritional science focused primarily on establishing associations between deficiency diseases and specific elements. Within this context, minerals were understood largely in terms of their presence or absence, rather than their chemical forms [3].

### 5.2. Analytical Constraints

Early analytical techniques (such as basic atomic absorption spectroscopy and flame photometry) were effective for quantifying total elemental content but were limited in their ability to discriminate among specific binding geometries and coordination states. As a result, research designs that did not differentiate mineral forms became standardized. To overcome these limitations, modern mineral research utilizes hyphenated and synchrotron-based analytical platforms to resolve specific chemical speciation within complex food matrices. Size-exclusion and hydrophilic interaction chromatography coupled to inductively coupled plasma mass spectrometry (SEC/HILIC-ICP-MS) enable non-destructive separation of macromolecular metalloproteins from low-molecular-weight chelates and free ions under native pH. Additionally, high-resolution electrospray ionization tandem mass spectrometry (ESI-HR-MS/MS) provides molecular-level validation of intact coordination stoichiometry before and after simulated gastrointestinal digestion. Furthermore, synchrotron X-ray absorption spectroscopy (XAS)-including XANES and EXAFS-serves as the definitive tool for in situ, non-destructive determination of central atom oxidation states, coordination numbers, and interatomic bond distances directly within intact food systems.

### 5.3. Experimental Standardization and the Use of Inorganic Salts

Inorganic salts were widely adopted as standard experimental materials due to their reproducibility and ease of quantification. In addition, the historical separation between nutritional and toxicological research traditions contributed to delays in the development of integrative approaches that consider mineral form [16]. This historical dichotomy has perpetuated the use of inorganic salts as default experimental reagents in basic biomedical studies, obscuring the distinct cellular transport pathways, membrane stability profiles, and metabolic handling mechanisms characteristic of physiological, food-bound coordination complexes.

## 6. Discussion

### 6.1. Conflation of Element-Based Effects and Form-Dependent Effects

The case studies reviewed herein illustrate how the physiological effects of minerals have often been generalized as intrinsic properties of the elements themselves. However, in many studies, the experimental materials employed were inorganic salts or ionic forms, and it remains insufficiently established whether the effects observed under such conditions are reproducible in the context of dietary intake or under complex physiological conditions governed by intact macromolecular matrices.

### 6.2. Differences in Biological Handling of Organic and Inorganic Mineral Forms

Minerals present in foods are ingested in association with organic matrices and may be released gradually during digestion or interact with specific transport and metabolic pathways. In contrast, minerals supplied as inorganic salts typically exist in ionic form in aqueous environments and may follow relatively rapid and nonspecific routes of absorption and excretion.

Although ionization or ligand exchange ultimately occurs during digestion, the binding form prior to ionization can influence release kinetics, competitive absorption, tissue redistribution, and recycling pathways, indicating that distinctions based on chemical form remain biologically meaningful. At the cellular level, these divergent kinetic inputs determine whether a trace element is safely buffered by intracellular chaperones and protein reservoirs or transiently elevates free cytosolic pools to induce oxidative stress, competitive transporter saturation, or non-specific enzyme inhibition.

### 6.3. Limitations of Binary Interpretations

Discussions surrounding organic and inorganic minerals have frequently focused on the question of which form is superior. However, biological systems should be understood as utilizing different mineral forms in complementary ways. Ionic inorganic minerals are essential for immediate transmembrane electrochemical events and acute signaling cascades, whereas minerals bound within organic matrices support sustained structural stability, controlled enzymatic delivery, and cellular metalloprotein synthesis (Figure 3).

Trafficking pathways, maintaining stable cellular bioenergetics and selenoprotein/metalloenzyme activity across a significantly broader safety window.

### 6.4. Reconsideration of Previous Interpretations in Mineral Research

Caution is warranted when extending findings obtained from studies using inorganic salts directly to the interpretation of effects associated with food-derived minerals. In particular, real-world nutritional conditions-such as long-term intake, chronic exposure, and sustained low-dose consumption-are not adequately represented by short-term supplementation studies using inorganic salts. Extrapolating the acute pharmacological responses of inorganic ions to long-term dietary recommendations risks mischaracterizing both physiological requirements and toxicological thresholds, as illustrated by the paradoxical outcomes observed in large-scale trace element trials.

### 6.5. Implications for Future Research Design

Future mineral research should adopt comparative designs that explicitly distinguish chemical forms for the same element.

In addition to short-term absorption, integrated evaluations encompassing long-term tissue distribution, accumulation, metabolic pathways, and physiological responses are needed. Modern experimental frameworks must incorporate speciation-resolved analytical techniques (such as LC-ICP-MS), targeted transcriptomic profiling of metal transporters, and organelle-specific bioenergetic assays to define the molecular boundaries between physiologic utilization and toxicity. Merging molecular toxicology with cellular nutrition will establish more precise, form-specific dietary and therapeutic paradigms.

## Figures and Tables

**Table 1:** Thermodynamic Stability Constants (log K), Predicted Gastrointestinal Speciation, and Mucosal Transport Pathways of Representative Mineral Forms. Classification simply as “organic” is chemically insufficient: weak organic acid salts dissociate extensively in the gastric lumen and rely on canonical inorganic permeases, whereas high-stability bidentate chelates (logβ_2_ > 7.5) and supramolecular matrices maintain coordinate integrity to engage alternative apical entry pathways and evade antagonist precipitation.

Mineral Form/Formulation Category	Representative Compound	Thermodynamic Stability Constant (logK or logβn)	Gastric Lumen Behavior (pH 1.5–2.0)	Duodenal/Jejunal Specific Ion & Ligand Exchange (pH 5.5–7.4)	Predominant Apical Mucosal Transport Mechanism	Susceptibility to Luminal Precipitation (Phytates/Phosphates)
Inorganic Mineral Salts	FeSO_4_, ZnSO_4_, ZnCl_2_, CaCO_3_, MgO	N/A (ionic lattices; complete ionization)	Instantaneous ionization into solvated, fully charged divalent cations (M^2+^)	Remain as unshielded free ions; undergo severe competition with endogenous/dietary ligands	Primary divalent ion permeases: DMT1 (SLC11A2), ZIP4 (SLC39A4), TRPV6	High (readily forms insoluble, unabsorbable precipitates)
Weak Organic Acid Salts	Zinc gluconate, Calcium lactate, Magnesium citrate	Low (log K ≈ 1.5–3.5)	Extensive acidolysis and proton-driven dissociation (M-L_n_ + nH^+^ = M^2+^ + nHL; >90% free cation release)	Dissociated cations behave identically to inorganic salts; weak ligands engage in non-protective exchange	Primary divalent metal permeases (DMT1, ZIP4); paracellular solvent drag	Moderate to High (minimal steric protection on post-dissociation)
High-Stability Bidentate Amino Acid Chelates	Zinc bisglycinate, Ferrous bisglycinate	High (logβ2 ≈ 7.5–8.5)	Coordination bonds largely resist gastric acidolysis; substantial intact coordinate fraction retained	Neutral charge shielding prevents precipitation; slow, buffered equilibrium exchange with enterocyte chaperones	Parallel pathways: intact PepT1 (SLC15A1) transport and/or fluid-phase endocytosis; canonical permeases post-cleavage	Low (steric hindrance and neutral charge minimize antagonist binding)
Endogenous Protein/Supramolecular Matrices	Ferritin nanocages, Casein phosphopeptides (CPPs)	Macromolecular assembly/Multi-site binding (log K ≈ 5.0–7.0)	Encapsulated cores resist complete acid hydrolysis; outer protein shells buffer inner mineral clusters	Gradual, enzymatic release via luminal proteases; soluble peptide-metal organophosphates prevent calcification	Macropinocytosis/receptor-mediated endocytosis (e.g., TfR1 for ferritin); passive paracellular diffusion for CPP complexes	Very Low (sequestration within macromolecular cavities shields against lattice precipitation)
Organometallic/Covalent Biomimetics	L-Selenomethionine (SeMet)	Covalent selenoether bond (not a coordination chelate)	Chemically stable across entire physiological pH range; unaffected by hydronium ion attack	Absorbed without structural alteration; acts as a structural mimic of essential amino acids	System L neutral amino acid transporters (LAT1/SLC7A5, LAT2/SLC7A8)	None (does not engage in ionic lattice reactions with mineral antagonists)

## Data Availability

No new data were created or analyzed in this study. Data sharing is not applicable to this article.
